# Association of a Self-Paid Medically Supervised Weight Management Program with Reversal of Obesity-Associated Impaired Fasting Glucose

**DOI:** 10.3390/clinpract11020053

**Published:** 2021-06-15

**Authors:** Vijaya Surampudi, Xinkai Zhou, Chi-Hong Tseng, David Heber, Zhaoping Li

**Affiliations:** 1 Center for Human Nutrition, University of California Los Angeles, 1000 Veteran Ave A670, Los Angeles, CA 90024, USA; vsurampudi@mednet.ucla.edu (V.S.); dheber@mednet.ucla.edu (D.H.); 2 Department of Medicine Statistic Core, University of California Los Angeles, Los Angeles, CA 90024, USA; XinkaiZhou@mednet.ucla.edu (X.Z.); ctseng@mednet.ucla.edu (C.-H.T.)

**Keywords:** obesity, impaired fasting glucose, prediabetes, weight loss, personalized weight management

## Abstract

Aims: The progression of prediabetes to T2DM can be delayed through diet modification and weight management. However, the intensive lifestyle program is often not covered by medical insurance. This retrospective analysis evaluates the association of a patient self-paid weight management program on an improvement of blood sugar in overweight and obese patients with impaired fasting glucose (IFG). Methods: The medical records of 4634 patients who participated in the self-pay UCLA Weight Management Program were reviewed and 2572 patients met the criteria for this retrospective analysis to examine whether this program was associated with the reversal of IFG over 3 months among 1396 patients with normal fasting glucose (NFG) and 1176 with IFG. Results: The patients with IFG lost comparable amounts of weight (10.5 ± 1.3 kg) at three months, as did the subjects with NFG (10.1 ± 1.3 kg). Fasting blood glucose in the IFG group decreased from 108.49 ± 6.4 to 101.8 ± 9.41 mg/dL (*p* < 0.0001) after three months. There were also significant reductions in triglycerides, and both systolic and diastolic blood pressure in both groups in association with weight loss. Conclusion: Our medically supervised self-pay multidisciplinary weight management program was associated with reduced fasting blood glucose levels in patients with IFG over three months with comparable weight loss to patients with NFG.

## 1. Introduction

Excess body fat, especially intra-abdominal fat, is central to the progression of prediabetes to Type 2 Diabetes Mellitus (T2DM). One measure of impaired glucose tolerance is impaired fasting glucose (IFG) defined as a fasting blood sugar >100 mg/dL. The Centers for Disease Control and Prevention (CDC) estimates 88 million, or more than a third of the U.S. population, to have prediabetes [1], which has been observed to progress to T2DM at a rate of 5–10% annually [2]. Obesity and prediabetes have also been associated with hypertension, lipid disorders, obstructive sleep apnea, fatty liver disease, and cancer [3]. Microvascular changes have been identified prior to the development of T2DM, including nephropathy, neuropathy, erectile dysfunction, and retinopathy [4]. Prediabetes and obesity are also associated with an increased risk of cardiovascular diseases [5,6]. A continuous inverse correlation between glucose levels and cognitive test results in the absence of diabetes has been demonstrated in cross-sectional population studies [7]. 

The most recent Endocrine Society clinical practice guidelines for the primary prevention of atherosclerotic cardiovascular disease and T2DM in patients at metabolic risk recommend prescribing lifestyle modification to reduce blood glucose prior to instituting a pharmacological treatment [8]. These recommendations are based on several hallmark trials, including the U.S. Diabetes Prevention Program (DPP) and Look AHEAD (Action for Health in Diabetes) study [9,10]. The DPP reported that lifestyle modification resulted in a 58 percent reduction in the incidence of T2DM and was more effective than metformin [9]. The Da Qing Diabetes Prevention Outcome Study in China demonstrated that lifestyle intervention in people with impaired glucose tolerance delayed the onset of T2DM and reduced the incidence of cardiovascular events, microvascular complications, and all-cause mortality [11].

Clinical guidelines and preventive strategies have been developed to be implemented in routine clinical settings. However, there is limited success in enrolling patient in lifestyle modification programs due to financial considerations, even though cost-effectiveness might be achieved at the population level [12,13]. This retrospective study analyzed the clinic records of patients enrolled in the multidisciplinary supported UCLA Medical Weight Management program to evaluate the association of a real-world patient self-supported program on the reduction in fasting glucose via weight reduction.

## 2. Methods

### 2.1. Subjects

This was a retrospective study of 4634 patients who participated in the UCLA Weight Management Program (also called the UCLA Risk Factor Obesity Program) between 1991 and 2017. The study consists of a retrospective review of medical records retained by the program and was approved by the Institutional Review Board of University of California, Los Angeles. Patient records were included in the analysis if patients met the following inclusion criteria: (1) >18 years of age; (2) overweight or obese with a BMI > 25 kg/m^2^; (3) enrolled in the program for the first time; (4) participated in the program weekly without missing more than three consecutive weekly visits during the three-month period. The patients with diagnosed T2DM, based on a fasting blood glucose > 126 mg/dL, or taking hypoglycemic medications were excluded from study. 

### 2.2. Clinical Program

The UCLA Medical Weight Management Program focuses on individualized dietary prescriptions based on lean body mass assessed using bioimpedance analysis (BIODYNAMICS BIA 450 bioimpedance analyzer, Shoreline, WA, USA). Typical meal plans included between 5–10 meal replacements/day and a total of 500 to 1000 calories/day. Meal replacement are products that are available for medically supervised weight loss programs, typically containing 15 g of protein per 100 calories, and utilized high quality protein combined with 40% carbohydrates, vitamins, and minerals. The proteins used included soy, pea, casein, and whey proteins. Most of the patients began the program with total meal replacement, while some selected a modified plan including whole food meals. The prescribed calories from the meal replacements and food were based on calorie deficits below the resting metabolic rate estimated from lean body mass. Patients were prescribed dietary protein intakes matched to their lean body mass at 1 g per pound of lean body mass per day, leading to a total protein intake of approximately 2.2 g of protein/kg body weight per day. After a period of time, many of the patients transitioned to a partial meal replacement program, where four or five servings of meal replacements per day were combined with a defined meal of about 300–500 calories of lean protein and non-starchy vegetables. After the first 2 weeks of the program, the patients were encouraged to exercise for 30 min per day and to add resistance exercises as tolerated. 

Weights and vital signs were measured weekly. A physician and dietitian evaluated all of the patients at beginning of the program and then once a month, or as needed. The program also included voluntary attendance at weekly group behavior change and nutrition education classes. Laboratory tests including complete blood count, electrolytes, and a basic metabolic panel (Quest Diagnostics, San Juan Capistrano, CA, USA) were also recorded at baseline and at 3-week intervals. The BIA to assess lean body mass was repeated every 6–8 weeks. 

The cost of the program varied from USD 50–65 per week for each patient depending on the level of dietary restriction and need for monitoring. Payments were collected monthly in advance and were non-refundable unless there was prior notice for planned vacations or emergencies. Laboratory tests, body composition analysis, and classes were included in the monthly program fee. 

### 2.3. Outcome Measures

The primary outcome was the amount of weight-loss and fasting blood glucose values after three months. The secondary outcomes included percent body fat, blood pressure, and lipid levels. 

### 2.4. Statistical Analysis 

Patient demographic data and baseline clinical history were summarized using the median (IQR) for continuous variables and frequency (percentage) and for categorical variables. Continuous variables were compared using the Wilcoxon rank-sum test, whereas categorical variables were compared using Pearson’s chi-squared test. We also summarized the number (percentage) of patients who remained in the study at three months.

Glucose levels were analyzed as a binary variable (IFG) if the levels were greater than 100 mg/dL. For the continuous glucose outcome, we used the linear mixed effects model with a fixed time and group (baseline IFG status Yes/No) effect and a random subject effect to model the trajectory of glucose levels over time. Specifically, time was modeled as piecewise linear with change points at 1 and 3 months. The random effects model takes into account the correlation between the observations within the same subject. In addition, we tested for the difference in glucose levels for 0 versus 3 months and reported the *p*-values.

For the binary IFG (Yes/No) outcome, we used the generalized linear mixed effects model with the logit link function to model the proportion of IFG patients over time. We also tested for the difference in proportions for 0 versus 3 months and reported the *p*-values. A *p*-value < 0.05 was considered statistically significant. R version 3.2.3 (www.r-project.org, last accessed on 17 May 2018) was used for the analyses. 

## 3. Results

### 3.1. Patient Population

**Figure 1:**
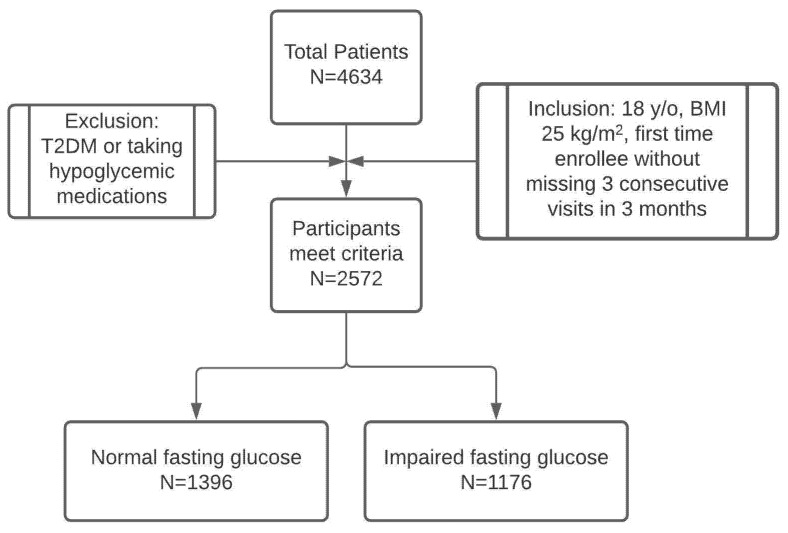
Patient chart review included in the study analysis.

There were 4634 unique patients enrolled in our program from 1991–2017. A total of 2572 met the inclusion and exclusion criteria and were included in the study (Figure 1). While patients were enrolled from anywhere from one month to 12 months in the program, we reviewed the data from the first three months of the program. The baseline characteristics of the patients are in Table 1. Among 2572 patients, a total of 1176 (73.1% female; 26.9% male) patients had IFG with a fasting blood glucose level between 100 and 125 mg/mL and 1396 patients (77.8% female; 22.2% male) were found to have an NFG level of <100 mg/dL. The patients were significantly older and had a greater BMI in the IFG group compared to the NFG group.

Other than appointment reminders for patients who missed two consecutive weekly appointments, there were no efforts made for the retention of participants. There were 843 patients remaining in the NFG group and 755 patients remaining in the IFG group at the end of 3 months. The patients who left the program and those who remained in the program but who missed three weekly visits for any reason were not included in the analysis. There were 421 patients left in the program at month three in the IFG group. 

### 3.2. Body Weight and Body Composition

**Figure 2:**
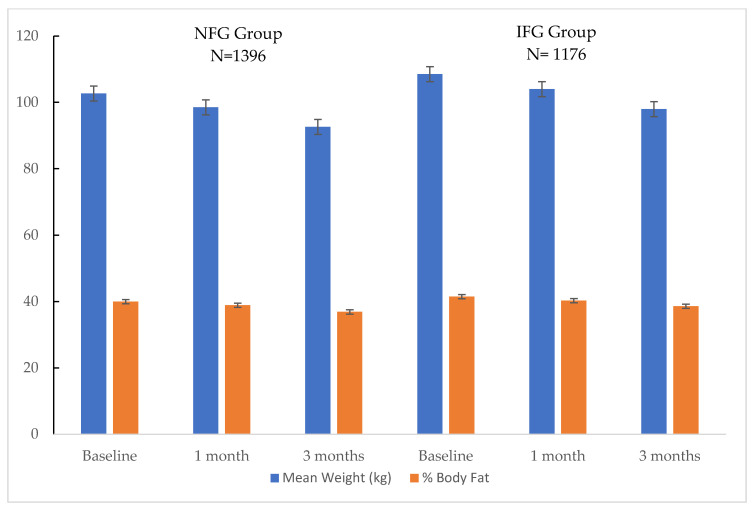
Change in weight (kg) and body fat (%) over the 3 months. Mean ± standard error. Dark blue bar: body weight. Orange bar: body fat.

The change in body weight is summarized in Figure 2. The median weight loss for the NFG group was 4.2 ± 1.3 kg at 1 month and 10.1 ± 1.3 kg at 3 months. The total weight loss for the IFG group was 4.5 ± 1.4 kg at 1 month and 10.5 ± 1.3 kg at 3 months. The IFG group patients had a statistically higher baseline weight than the NFG group patients. However, there was no significant difference in the weight loss between the groups at any point of time. 

The effect of the program on body composition is summarized in Figure 2. The average body fat percentage using BIA at baseline was 41.5 ± 0.9% for the IFG group and 40 ± 0.8% for the NFG group (*p* < 0.05). However, there was no significant difference in the reduction in body fat percentage between the above two groups of patients over 3 months.

### 3.3. Fasting Blood Glucose

The average blood glucose level for the IFG group of patients at baseline was 108.49 ± 6.4 mg/dL, with a statistically significant decrease to 101.8 ± 9.41 mg/dL (*p* < 0.0001) at 3 months. The fasting blood glucose level for the NFG group at entry was 89.8 ± 6.61 mg/dL, with no significant change at 3 months with fasting glucose levels of 91.1 ± 8.5 mg/dL (Table 2).

**Figure 3:**
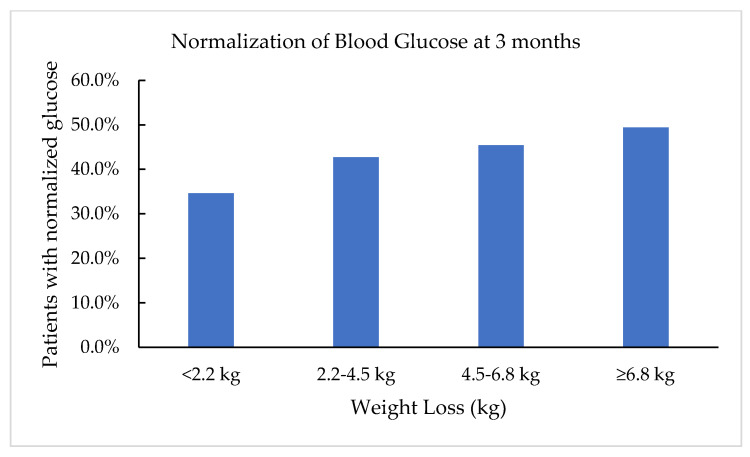
Percentage of patients with normalization of blood glucose levels <100 mg/dL in relation to amount of weight lost at 3 months.

After three months, over 40% of the patients who lost 2.2–4.5 kg was able to normalize their fasting blood glucose levels. Within the group of patients who were able to lose more than 6.8 kg, nearly 50% of the patients normalized their blood glucose levels (Figure 3).

### 3.4. Lipids

The baseline total cholesterol was comparable in all of the patients. The triglyceride level for the IFG patients was significantly decreased at 3 months by 45.2 ± 64.2 mg/dL (*p* < 0.0001). The high-density lipoprotein cholesterol level for the patients in both groups was significantly increased in association with weight loss, but without any significant difference between the groups. There was a significant decrease in low-density lipoprotein at 3 months by 10.9 ± 29.45 mg/dL (*p* < 0.0001) in the IFG group and by 12.8 ± 31.6 (*p* < 0.0001) in the group with normal fasting blood glucose (Table 2).

### 3.5. Blood Pressure

The IFG group had a statistically higher systolic and diastolic blood pressure at baseline compared to the group with NFG. The patients with IFG had a higher median, systolic blood pressure at baseline of 127.2 ± 1.1 mmHg compared to the NFG group patients (123.8 ± 1.1 mmHg, *p* < 0.001). There was a significant reduction in the systolic blood pressure at 3 months in both groups compared to the baseline (IFG patients, 121.3 ± 1 mmHg *p* < 0.001 and NFG patients, 118.9 ± 1 mmHg *p* < 0.001).

The patients with IFG also had a higher diastolic blood pressure at baseline (IFG patients, 81.9 ± 2.6 mmHg vs. the NFG group, 79.5 ± 2.4 mmHg, *p* < 0.001). There was a significant reduction in the diastolic blood pressure at 3 months in both groups compared to the baseline (IFG patients, 77.3 ± mmHg, *p* < 0.001 and NFG patients, 75.5 ± 2 mmHg, *p* < 0.001) (Table 2).

## 4. Discussion

This retrospective analysis demonstrated that a patient self-paid program was effective to help patients lose weight and improve fasting blood glucose effectively. In addition, the program was able to improve other cardiovascular health parameters, such as blood pressure (systolic and diastolic), and lipids (total cholesterol, HDL, LDL, and triglycerides). 

Our analysis has demonstrated that this self-pay program could be a viable and economically feasible approach to delay or prevent the development of type 2 diabetes and cardiovascular diseases in patients who are overweight or obese. Our cost-effective ambulatory care model enabled physicians to partner with dietitians, nurses, and behavior therapists by utilizing group nutrition and behavioral education in a weekly schedule, coordinating with time for patients to consult with physicians and dietitians individually, and reviewing laboratory data supporting the hypothesis that glucose monitoring improves weight loss outcomes [14]. There is no net cost to the healthcare group because this is a service entirely funded by monthly patient fees. The scale of the results presented in this paper could have significant potential for far-ranging economic and social impact, helping to alleviate the growing economic burden of treating IFG, and T2DM. 

The economic burden associated with T2DM and cardiovascular diseases is substantial for patients, employers, and healthcare systems. Research conducted by the American Diabetes Association has estimated the cost of diagnosed diabetes in 2017 as USD 327 billion, including USD 237 billion in direct medical costs and USD 90 billion in reduced economic productivity. The indirect costs include increased absenteeism (USD 3.3 billion) and reduced productivity while at work (USD 26.9 billion) for the employed population, reduced productivity for those not in the labor force (USD 2.3 billion), inability to work as a result of disease-related disability (USD 37.5 billion), and lost productive capacity due to early mortality (USD 19.9 billion) [15]. Weight management programs can reduce the development of T2DM if such programs can be cost-effective and efficient within our existing medical care systems [16]. Patient-orientated interventions are the most effective in effecting positive behavioral and health outcomes [17]. The impact of weight loss on medication utilization cannot be understated, even though the discontinuation of medications may not have the greatest impact on overall healthcare costs. However, the elimination of medications may be associated with a reduction in potential side effects and an improvement in quality of life.

The most significant comorbid disease associated with the global epidemic of obesity is T2DM, which is now linked to congestive heart failure, liver disease, and chronic renal failure. All of these disorders are driven by abdominal visceral and hepatic excess fat in both overweight and obese patients. While obesity with a BMI >30 kg/m^2^ has been declared a disease by the American Medical Association [18], many individuals around the world develop diabetes at a lower body mass index [19]. In the United States, women at a BMI of 27 kg/m^2^ have an increased risk of T2DM, and the overall risk at a BMI of 30 kg^/^m^2^ is 3000%, or thirty-fold. Although heart disease, hypercholesterolemia, gallstones, and other comorbid conditions have a four- to six-fold increased risk, T2DM is linked to obesity in an intimate way that has been termed ‘Diabesity’. In our outpatient program, we previously showed that obese, pre-diabetic, and T2DM patients all lost weight as effectively with very low calorie diet (VLCD) or low calorie diet (LCD) over 12 months [20].

A study of 377 patients scattered across Italy and treated by general physicians trained to safely and effectively prescribe a very-low carbohydrate ketogenic (VLCK) diet in clinical practice, reduced fasting plasma glucose (−8.7 ± 15.3 mg/dL, *p* < 0.001) in combination with average weight losses of 5 ± 3 kg (*p* < 0.001) at 12 weeks, with no changes to one year of follow-up [21]. 

A systematic review has examined the glycemic benefits of commercial weight-loss programs as compared to control/education or counseling among overweight and obese adults who had, or who were at an increased risk of, T2DM [22]. Among 18 randomized clinical trials selected systematically, few examined whether commercial weight-loss programs result in glycemic benefits for their participants, particularly among individuals at an increased risk of T2DM, with only suggestive trends. 

The CDC estimated that among the 29.1 million U.S. adults with T2DM, 8.1 million remained undiagnosed [23]. The increased incidence of obesity occurs in close geographic conjunction with an increase in the prevalence of T2DM and obligates a rapidly growing healthcare expenditure for T2DM management [24,25]. The Diabetes Prevention Program (DPP) multicenter trial clearly demonstrated that overweight and obese adults with pre-diabetes maintaining a weight loss of 2.5 kg at 2 years through lifestyle intervention significantly reduced their risk of developing T2DM. While the DPP trial demonstrated the benefits of weight loss on glycemia, achieving similar results in the community has been more challenging given the characteristics of individuals willing to participate in a clinical trial. The DPP used very intensive lifestyle interventions. On the other hand, commercial weight-loss programs similar to our program are common across the U.S. [26]. The major difference from commercial programs is that our self-pay program is based in a medical center utilizing clinical space at times when it is not being used for other clinical programs. A systematic review of leading commercial weight loss programs evaluated only weight loss, adherence, and the harm of commercial or proprietary weight loss programs to control/educate or behavioral counseling in 39 RCTs among overweight and obese adults and did not document changes in fasting plasma glucose [27]. 

We acknowledge that this study utilized a retrospective review of a medical records and has some significant limitations. There were significant numbers of patients who did not attend the program for 3 months. Medications were not captured in the record and we may have underestimated the effects of weight loss on glucose control medications as a result. Prediabetes was defined by impaired fasting blood glucose values instead of oral glucose tolerance tests or hemoglobin A1c. In addition, the data analysis did not examine pharmacotherapy for weight management, which was added as necessary to these strategies in a small subgroup of the patient population. 

## 5. Conclusions

This retrospective analysis of this self-pay multidisciplinary weight management program demonstrates a cost-effective option for healthcare groups to incorporate into primary care. The space utilized can be clinic space that is free in the evenings or at weekends. Given the low cost and part-time utilization of healthcare professionals in the program, this program could be practically applied in ambulatory care settings to treat prediabetes and delay or prevent the onset of T2DM. 

## Figures and Tables

**Table 1:** Demographic Information.

	Normal Fasting Glucose (<100 mg/dL)	Impaired Fasting Glucose (100–126 mg/dL)	*t*-Test *p*-Value
Patient number	1396	1176	–
Variables	Median (IQR)	Median (IQR)	–
Age (years)	55.7 (45.4, 64.4)	61.1 (51.8, 68.5)	<0.001
Weight (kg)	99.6 (85.4, 118)	105.8 (90.1, 126.7)	<0.001
BMI (kg/m^2^)	35.2 (31.1, 40.7)	37.2 (32.7, 43.0)	<0.001
Gender: Male	310 (22.2%)	316 (26.9%)	0.007

**Table 2:** Blood glucose and lipid levels.

Variable	Normal Fasting Glucose			Impaired Fasting Glucose		
	Baseline	3 Months	*p*-Value (Compared to Baseline)	Baseline	3 Months	*p*-Value (Compared to Baseline)
Subjects (N)	1396	843	–	1176	755	–
SBP (mmHg)	123.8 ± 11.6	118.9 ± 11.7	<0.001	127.2 ± 12.2	121.3 ± 11.3	<0.001
DBP (mmHg)	79.5 ± 7.2	75.5 ± 6.9	<0.001	81.9 ± 7.4	77.3 ± 6.9	<0.001
Glucose (mg/dL)	89.77 ± 6.61	91.12 ± 8.50	0.15	107.49 ± 6.40	101.29 ± 9.41	<0.001
LDL (mg/dL)	180.13 ± 37.85	167.86 ± 33.20	<0.001	179.97 ± 41.20	169.68 ± 36.76	<0.001
Triglycerides (mg/dL)	138.50 ± 84.06	104.28 ± 57.26	<0.001	161.63 ± 84.36	119.27 ± 58.83	<0.001
HDL (mg/dL)	60.31 ± 16.25	56.21 ± 14.60	<0.001	56.95 ± 13.52	53.32 ± 12.30	<0.001

## Abbreviations

BIA	bioimpedance analysis
BMI	body mass index
DPP	diabetes prevention program
HDL	high-density lipoproteins
IFG	impaired fasting glucose
LCD	low calorie diet
LDL	low-density lipoproteins
NFG	normal fasting glucose
VLCD	very low calorie diet
T2DM	type 2 Diabetes Mellitus
